# Parsing brain-behavior heterogeneity in very preterm born children using integrated similarity networks

**DOI:** 10.1038/s41398-023-02401-w

**Published:** 2023-04-03

**Authors:** Laila Hadaya, Konstantina Dimitrakopoulou, Lucy D. Vanes, Dana Kanel, Sunniva Fenn-Moltu, Oliver Gale-Grant, Serena J. Counsell, A. David Edwards, Mansoor Saqi, Dafnis Batalle, Chiara Nosarti

**Affiliations:** 1 Centre for the Developing Brain, Department of Perinatal Imaging and Health, Faculty of Life Sciences & Medicine, King’s College London, London, UK; 2 Department of Child and Adolescent Psychiatry, Institute of Psychiatry Psychology and Neuroscience, King’s College London, London, UK; 3 Translational Bioinformatics Platform, NIHR Biomedical Research Centre, Guy’s and St. Thomas’ NHS Foundation Trust and King’s College London, London, UK; 4 Centre for Neuroimaging Sciences, Institute of Psychiatry Psychology and Neuroscience, King’s College London, London, UK; 5 Department of Forensic and Neurodevelopmental Sciences, Institute of Psychiatry Psychology and Neuroscience, King’s College London, London, UK; 6 MRC Centre for Neurodevelopmental Disorders, King’s College London, London, UK

**Keywords:** Predictive markers, Neuroscience

## Abstract

Very preterm birth (VPT; ≤32 weeks’ gestation) is associated with altered brain development and cognitive and behavioral difficulties across the lifespan. However, heterogeneity in outcomes among individuals born VPT makes it challenging to identify those most vulnerable to neurodevelopmental sequelae. Here, we aimed to stratify VPT children into distinct behavioral subgroups and explore between-subgroup differences in neonatal brain structure and function. 198 VPT children (98 females) previously enrolled in the Evaluation of Preterm Imaging Study (EudraCT 2009-011602-42) underwent Magnetic Resonance Imaging at term-equivalent age and neuropsychological assessments at 4–7 years. Using an integrative clustering approach, we combined neonatal socio-demographic, clinical factors and childhood socio-emotional and executive function outcomes, to identify distinct subgroups of children based on their similarity profiles in a multidimensional space. We characterized resultant subgroups using domain-specific outcomes (temperament, psychopathology, IQ and cognitively stimulating home environment) and explored between-subgroup differences in neonatal brain volumes (voxel-wise Tensor-Based-Morphometry), functional connectivity (voxel-wise degree centrality) and structural connectivity (Tract-Based-Spatial-Statistics). Results showed two- and three-cluster data-driven solutions. The two-cluster solution comprised a ‘resilient’ subgroup (lower psychopathology and higher IQ, executive function and socio-emotional scores) and an ‘at-risk’ subgroup (poorer behavioral and cognitive outcomes). No neuroimaging differences between the resilient and at-risk subgroups were found. The three-cluster solution showed an additional third ‘intermediate’ subgroup, displaying behavioral and cognitive outcomes intermediate between the resilient and at-risk subgroups. The resilient subgroup had the most cognitively stimulating home environment and the at-risk subgroup showed the highest neonatal clinical risk, while the intermediate subgroup showed the lowest clinical, but the highest socio-demographic risk. Compared to the intermediate subgroup, the resilient subgroup displayed larger neonatal insular and orbitofrontal volumes and stronger orbitofrontal functional connectivity, while the at-risk group showed widespread white matter microstructural alterations. These findings suggest that risk stratification following VPT birth is feasible and could be used translationally to guide personalized interventions aimed at promoting children’s resilience.

## Introduction

Very preterm birth (VPT; ≤32 weeks’ gestation) is associated with an increased likelihood of developing cognitive and behavioral difficulties across the lifespan [1–5]. Efforts to conceptualize these difficulties have proposed a “preterm behavioral phenotype”, characterized by problems in emotional and social processing, and inattention [6]. However, while some VPT children display a behavioral profile reflecting a preterm phenotype, others follow typical developmental trajectories [7–9]. Such behavioral heterogeneity following VPT birth presents a challenge for building risk prediction models [10], as multiple causes may lead to the same outcome and as a single mechanism may lead to multiple outcomes [11].

Several endogenous and exogenous factors contribute to a child’s behavioral development and a complex interplay between environmental, clinical, and neurobiological features could result in co-occurring neurodevelopmental, cognitive and behavioral difficulties following VPT birth [12]. These factors are often non-independent and their combination (e.g., neurobiological and socio-demographic variables) may result in improved prediction of functional outcomes [13]. For instance, both socio-demographic deprivation and increased neonatal clinical risk have been associated with neurodevelopmental as well as behavioral difficulties in VPT children. These encompass executive and socio-emotional functions [14–16], which could be considered as gateway mechanisms that shape behavioral outcomes, as they are subserved by brain networks relating to both bottom-up stimulus processing and top-down behavioral control [17]. Impairments in these domains have in fact been associated with later academic and mental health difficulties [3, 18].

Previous studies have attempted to stratify outcome heterogeneity in preterm children using clustering and latent-class analyses [7–9, 19, 20]. These studies typically used cognitive and behavioral measures as input features, and then compared subgroups in terms of specific clinical and environmental risk factors that were not used in the stratification analyses (i.e., out-of-model). Some found differences in neonatal clinical profiles between subgroups of preterm children [20] and others showed that familial characteristics, such as parental education, maternal distress, and cognitively stimulating parenting, differentiated resilient subgroups from those exhibiting behavioral difficulties [8, 9]. Here, instead, we chose to include input measures of known risk factors (i.e., clinical and environmental variables) alongside in-model cognitive and behavioral measures, in order to delineate the complex interplay between different risk factors and behavioral outcome measures; thus increasing the likelihood of discovering nuanced subtypes of preterm children who exhibit similar behavioral outcomes, but with possibly different underlying correlates (i.e., equifinality) [11].

A growing body of research, investigating specific factors associated with later behavioral outcomes, is studying the early neural signatures that may shape an individual’s neurodevelopmental trajectory. Alterations in brain volumes [21, 22], white matter microstructure [23, 24], and functional connectivity [25, 26] at birth in regions and networks subserving social, emotional and attentional processes, have been associated with later behavioral difficulties in VPT samples. Differences between latent subgroups of VPT children and infants have been previously studied in relation to qualitative measures of brain abnormalities and/or high grade brain injury based on neonatal Magnetic Resonance Imaging (MRI), as well as quantitative differences in brain tissue volumes [8, 27, 28]. However, it remains to be explored whether distinct multidimensional subgroups of VPT children could also be characterized by localized differences in early brain development using advanced quantitative measures of brain structure and function, such as log-Jacobians, tract based spatial statistics and degree centrality, which have previously been used in neonatal samples [29–31]. Conducting analyses at the whole-brain and voxel-wise level, allows for an enhanced spatial localization of potential structural and functional between-subgroup differences, thus extending previous research [8, 27, 28].

The main aim of this study was to parse brain-behavior heterogeneity in VPT children, by identifying subgroups with similar environmental, clinical and behavioral profiles and examining between-subgroup differences in structural and functional brain features at term-equivalent age. Firstly, we implemented an integrative clustering approach (Similarity Network Fusion; SNF) [32] to stratify VPT children into distinct subgroups based on three data types: (i) neonatal clinical and socio-demographic variables, (ii) childhood socio-emotional outcomes and (iii) executive function measures. The advantage of this approach is that it integrates sample-similarity networks built from each distinct data type and constructs a final integrated network, which contains common and complementary information from the different data types. This is then used to stratify the sample into distinct subgroups using clustering [32]. We also investigated whether resultant subgroups differed in outcomes that were not used in stratification analyses (i.e., out-of-model variables); in order to provide external validation [33–35]. Finally, we explored between-subgroup differences in regional brain volume and structural and functional connectivity at term-equivalent age. We hypothesized that there would be distinct subgroups of VPT children characterized by unique neonatal neural signatures.

## Methods

### Study design

#### Participants

Five hundred and eleven infants born VPT were recruited from 14 neonatal units in London in 2010–2013 and entered the Evaluation of Preterm Imaging Study (ePrime; EudraCT 2009-011602-42) [36]. Infants with congenital malformation, prior MRI, metallic implants, whose parents did not speak English or were subject to child protection proceedings were not eligible for participation in the study.

Participants underwent multimodal MRI at 38–53 weeks post-menstrual age (PMA) on a 3-Tesla MR imaging system (Philips Medical Systems, Best, The Netherlands) located on the neonatal intensive care unit at Queen Charlotte’s and Chelsea Hospital, London, using an 8-channel phased array head coil. For data acquisition and imaging parameters see Supplemental Information. Infants whose parents chose sedation for the procedure (87%) received oral chloral hydrate (25–50 mg/kg).

In total, 251 participants (including 29 sets of multiple pregnancy children) were followed-up between the age of 4 and 7 years at the Center for the Developing Brain, St Thomas’ Hospital, London. This was a convenience sample corresponding to 82% of 306 participants who were past their fourth birthday by the study end date, September 1^st^ 2019, and had consented to be contacted for future research. Invitations for follow-up were sent in chronological order of birth.

Ethical approval was granted by the Hammersmith and Queen Charlotte’s Research Ethic Committee (09/H0707/98) and the Stanmore Research Ethics Committee (14/LO/0677). Informed consent was obtained from all participants.

#### Clinical and socio-demographic data

We used Principal Component Analysis (PCA) to select neonatal clinical variables of interest from a set of 28 available variables. These were: gestational age (GA) at birth, number of days on mechanical ventilation, number of days on continuous positive airway pressure (CPAP) and number of days on parenteral nutrition (TPN), which loaded onto a single component explaining 72% of the variance in the data. This component was labeled ‘neonatal sickness index’. Please refer to our previous work [24] and Supplemental Information for more details on the PCA analysis.

Socio-demographic risk was evaluated using a postcode derived measure of deprivation in England, the Index of Multiple Deprivation 2010 (IMD; http://tools.npeu.ox.ac.uk/imd/), whereby higher IMD scores reflect greater deprivation. The IMD combines neighborhood-specific information about seven domains of deprivation: income, employment, education/skills/training, health, crime, housing and living environment. The IMD was collected at the term-equivalent age. Continuous IMD scores were used in the integrative-clustering and evaluation of subgroup profile analyses. IMD quintiles are provided when reporting sample characteristics (Table 1) for ease of interpretability.

**Table 1:** Socio-demographic and clinical participant data.

		Integrative clustering sample *n* = 198	Diffusion MRI TBSS analysis sample *n* = 166	Structural MRI log-Jacobian analysis sample *n* = 165	rs-fMRI degree centrality analysis sample *n* = 129
Corrected age at assessment, years	Median	4.63	4.60	4.59	5.63
	Range	4.18–7.17	4.18–7.17	4.18–7.17	4.18–7.17
PMA, weeks	Median	42.57	42.43	42.57	42.43
	Range	38.29–52.86	38.29–44.86	38.29–44.86	38.29–44.86
Sex, male:female	n=	100:98	88:78	86:79	68:61
Self-reported maternal ethnicity	n (%)				
Asian		50 (25.3%)	44 (26.5%)	43 (26.1%)	34 (26.4%)
Black/African/Caribbean/Black British		30 (15.2%)	23 (13.9%)	25 (15.2%)	15 (11.6%)
Mixed/Multiple ethnic groups		3 (1.5%)	3 (1.8%)	3 (1.8%)	3 (2.33%)
White		112 (56.6%)	93 (56.0%)	91 (55.2%)	75 (58.1%)
Self-reported paternal ethnicity	*n* (%)				
Asian		34 (17.2%)	29 (17.5%)	27 (16.4%)	23 (17.8%)
Black/African/Caribbean/Black British		23 (11.6%)	19 (11.5%)	20 (12.1%)	14 (10.9%)
Mixed/Multiple ethnic groups		2 (1.0%)	1 (0.6%)	1 (0.6%)	0 (0.0%)
White		95 (48.0%)	80 (48.2%)	79 (47.9%)	63 (48.8%)
Neonatal IMD, quintiles	*n* (%)				
1 (least deprived)		49 (24.8%)	40 (24.1%)	38 (23.0%)	30 (23.3%)
2		37 (18.7%)	31 (18.7%)	32 (19.4%)	25 (19.4%)
3		44 (22.2%)	39 (0.6%)	38 (23.0%)	30 (23.3%)
4		48 (24.2%)	39 (23.5%)	38 (23.0%)	31 (24.0%)
5 (most deprived)		20 (10.1%)	17 (10.2%)	19 (11.5%)	13 (10.1%)
GA at birth, weeks	Median	30.14	30.29	30.14	30.14
	Range	23.86–32.86	24.00–32.86	24.00–32.86	24.00–32.86
Neonatal clinical risk	*n*=				
Days TPN, ratio 0:1:2		68:98:32	62:78:26	63:77:25	49:61:19
Days CPAP, ratio 0:1:2		33:125:40	30:107:29	31:103:31	23:82:24
Days ventilation, ratio 0:1:2		101:74:23	92:59:15	92:58:15	72:46:11

Note: Table describing sample socio-demographic and clinical characteristics for the integrative clustering and MRI analyses.

Neonatal clinical risk categories (0, 1 and 2) respectively correspond to zero days, more than zero days, but less than the top quintile, and within the top quintile. IMD quintiles 1–5 respectively correspond to the least deprived quintile (1) to the most deprived quintile (5). Ethnicity was grouped according to the Office of National Statistics classifications 2016 (see Supplementary Information).

*CPAP* continuous positive airway pressure, *GA* gestational age at birth, *IMD* Index of Multiple Deprivation, *PMA* post-menstrual age at scan, *rs-fMRI* resting-state functional MRI, *TBSS* Tract Based Spatial Statistics, *TPN* total parenteral nutrition.

#### Childhood assessment

Intelligence quotient (IQ) was evaluated using the Wechsler Preschool and Primary Scale for Intelligence (WPPSI-IV) [37] and executive function using the preschool version of the parent-rated Behavior Rating Inventory of Executive Function (BRIEF-P) [38]. Socio-emotional processing was evaluated using the Empathy Questionnaire (EmQue) [39] and the Social Responsiveness Scale, Second Edition (SRS-2) [40]. Psychopathology was assessed using the Strengths and Difficulties Questionnaire (SDQ) [41], temperament using the Child Behavioral Questionnaire - Very Short Form (CBQ) [42] and cognitively stimulating home environment using an adapted version of the Cognitive Stimulating Parenting Scale (CSPS) [43].

#### Exclusions

Twenty-seven participants were excluded due to incomplete childhood outcome data, 17 due to major brain lesions (periventricular leukomalacia, parenchymal hemorrhagic infarction, or other ischemic or hemorrhagic lesions), detected on neonatal T2-weighted MRI images at term by an experienced perinatal neuroradiologist, and 5 participants due to missing T2-weighted MRI images, hence the inability to evaluate the presence of major lesions (Fig. S1).

### Data integration and clustering

Analyses were conducted in R (version 3.6.1). Using SNF, three data types were integrated: (Type 1) neonatal socio-demographic and clinical variables: IMD at birth, GA, days on ventilation, days on TPN and days on CPAP. (Type 2) childhood socio-emotional outcomes: EmQue subscale raw scores - emotion contagion, attention to others’ emotions, prosocial behaviors and SRS-2 total raw score. (Type 3) childhood executive function: BRIEF-P raw subscale scores - inhibit, shift, emotional control, working memory and plan/organize.

Prior to integration, participants with in-model outlier values greater than 3 times the interquartile range were excluded. A total of 198 children were included in the SNF analyses. Zero-inflated neonatal clinical risk variables (days ventilation, days TPN and days CPAP) were converted into ordinal categorical variables with three levels: (Level 0: zero days; Level 1: greater than zero and not within the top quintile; Level 2: within the top quintile). For the mixed data type (numeric and categorical data; data type 1), Gower’s standardization based on the range was applied using the *daisy* function from cluster R package [44] and for numeric only matrices (data types 2 and 3), variables were standardized to have a mean value of 0 and a standard deviation of 1 using the *standardNormalization* function from SNFtool R package [45].

**Fig. 1:**
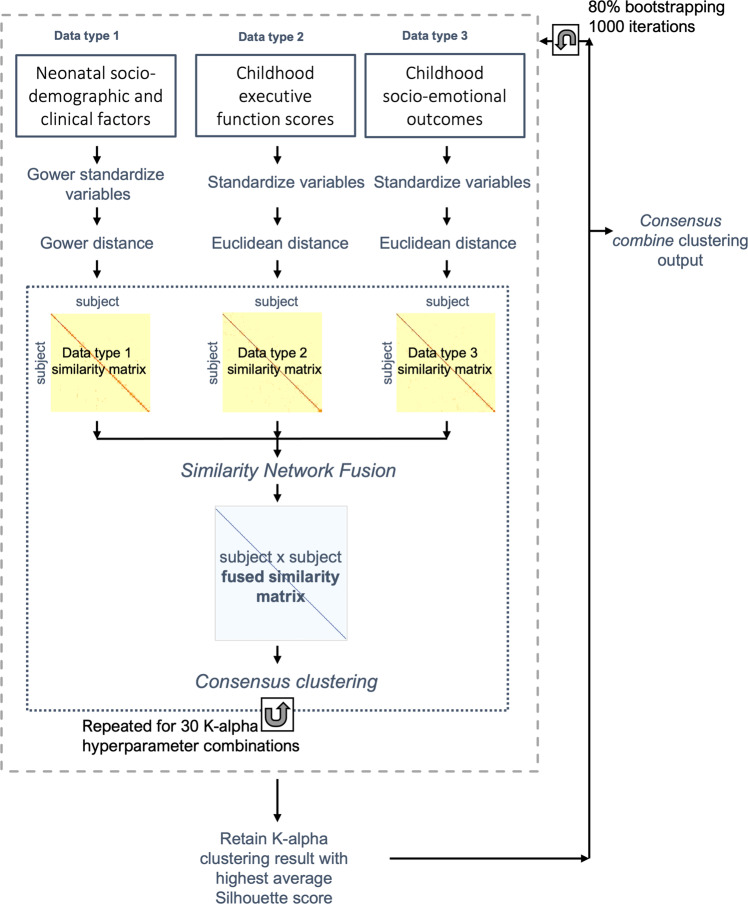
Data integration and clustering pipeline. Figure summarizing the data pre-processing (variable normalization), data integration and clustering pipeline executed in order to obtain the final consensus cluster assignment.

An adaptation of the *ExecuteSNF.CC* function [46] was used for the data integration and clustering steps. Dissimilarity Gower distance (for the mixed data type) and Euclidean distance (for numeric data types) matrices were calculated and used to create similarity matrices using the SNFtool R package’s *affinityMatrix* function [45]. This was followed by an integration of the similarity matrices using SNFtool’s *SNF* algorithm resulting in a ‘fused similarity matrix’ [45]. The integrative clustering process can be summarized into two steps:

Step 1: SNF method has two main hyperparameters, K and alpha. K (i.e., neighborhood size) indicates the number of neighbors of a node to consider when the similarity networks are being generated and alpha is an edge weighting parameter determining the weight of edges between nodes in the networks. We tried 30 combinations of K and alpha hyperparameters {K = 10, 15, 20, 25, 30; alpha = 0.3, 0.4, 0.5, 0.6, 0.7, 0.8}, similar to the approach followed in [47]. The K-alpha hyperparameter values were chosen based on the ranges recommended in the SNFtool R package, 10–30 for K and 0.3–0.8 for alpha [32, 45]. Consensus clustering, using *ConsensusClusterPlus* function [48], was then applied to each fused similarity matrix, corresponding to a K-alpha combination, where spectral clustering was run 1000 times with 80% of the population randomly subsampled for each clustering run and a single consensus clustering result obtained from hierarchical clustering. Step 2: Next, out of the 30 clustering results produced in step 1, the one with the highest average silhouette width score was retained. Steps 1 and 2 were repeated 1000 times in a bootstrap approach, after selecting and pre-processing the three data matrices of 80% of the sample set. The 1000 resultant retained clustering outputs were then fed to the diceR R package’s *consensus_combine* function [49] which implements hierarchical clustering on the consensus matrix and generates the final consensus clustering. Figure 1 summarizes the data-integration and clustering steps and the code used can be accessed here: https://github.com/lailahadaya/preterm-ExecuteSNF.CC. Further details can also be found in Supplemental Information.

Before implementing steps 1 and 2, it was essential to determine the number of clusters. For this, we used the SNFtool R package’s *estimateNumberOfClustersGivenGraph* function [45] to calculate Eigengap and Rotation Cost heuristics for each K-alpha combination (Fig. S2). This process suggested C = 2, C = 3 and C = 4 as the optimal number of clusters. Consensus matrices and silhouette scores were generated and compared for these three potential clustering solutions (Fig. S2). Resultant subgroups from C = 2 and C = 3 were chosen to be evaluated for phenotypic differences, as their silhouette scores and consensus matrices gave better values in comparison to those of C = 4 (Fig. S2). More details on the estimation of cluster numbers can be found in Supplemental Information. An alluvial plot was used to illustrate the transition of subject subgroup classification between the two-cluster and three-cluster solutions (Fig. S3).

### Evaluation of subgroup profiles

Resultant subgroups were characterized based on in-model and out-of-model variables. For the out-of-model features, subgroups were compared in terms of psychiatric symptoms (SDQ internalizing, externalizing problems and total scores), temperament (CBQ negative affectivity, surgency and effortful control scores), cognitive abilities (WPPSI full-scale IQ), and cognitive stimulation at home (CSPS score). Details on selection of in-model and out-of-model variables can be found in Supplemental Information and Figs. S4 and S5.

For numeric measures, between-subgroup differences were assessed using non-parametric one-way tests: Mann-Whitney when C = 2 or Kruskal Wallis when C = 3 [50]. Shapiro-Wilk test was used to assess normality. For categorical variables, Chi-squared test was used to evaluate differences in proportions of individuals in each group when count per cell was >5 and Fischer’s Exact test was used otherwise. To compare differences between the ordinal neonatal clinical variables with 3 categories (Levels 0, 1 and 2) and the non-ordinal subgroups from C = 2 and C = 3, the Extended Cochran-Armitage Test was used. We also ran supplementary post-hoc analyses investigating subgroup differences in clinical variables not included as in-model variables (please see Supplementary Information for more details).

Results with *p* < 0.05 were considered to be statistically significant. To correct for multiple comparisons the False Discovery Rate method was used. The same statistical analyses were repeated using general linear models correcting for potential confounders (age and sex) and 5000 permutation test iterations [51]. Effect sizes for non-normally distributed variables were measured using Wilcoxon Glass Rank Biserial Correlation (gr) for measuring differences between two groups and Epsilon Squared for three groups. For continuous normally distributed variables, Cohen’s F was used and Cramer’s V for categorical variables.

### Exploring neonatal brain differences between subgroups

Tract Based Spatial Statistics (TBSS) was used to assess white matter microstructure at the voxel-level using fractional anisotropy (FA) and mean diffusivity (MD) maps [52]. FA approximates the directional profile of water diffusion in each voxel and MD measures the average movement of water molecules within a voxel. Higher FA and lower MD values reflect more optimal white matter myelination and microstructure. For diffusion MRI (d-MRI) image pre-processing and TBSS protocol details please refer to Supplemental Information.

Structural MRI (s-MRI) log-Jacobian determinant maps were calculated to quantify regional brain volumes (greater log-Jacobian values reflect larger relative structural volumes), using Tensor Based Morphometry, following methods described in our previous work [53, 54] and in Supplemental Information.

Resting-state functional MRI (rs-fMRI) data were pre-processed as in our previous work;[55] for more details see Supplemental Information. Functional connectivity was evaluated using a measure of weighted degree centrality at the voxel-level (i.e., the sum of the correlations between the time-series of each voxel and all other voxels within a gray matter mask of the brain) [31, 56]. Edges with a correlation coefficient below a threshold of 0.2 were excluded and the degree centrality values for each voxel in the gray matter mask were z-scored and used in subsequent between-subgroup analyses. Whilst other functional network measures are available (i.e., participation coefficient and within module-z [57], we opted to study degree centrality as we recently showed this to be disrupted in preterm born neonates [31]. Furthermore, degree centrality is a good voxel-wise summary measure of connectivity strength, which is reliable and correlates with relevant phenotypes, such as age and sex [58]. It has been used to study typical cognitive function [59] and has recently been shown to be a reproducible metric to detect atypical functional connectivity patterns in neurodevelopmental disorders [56].

The number of children included in the different modality-specific MRI analyses slightly differed due data availability: TBSS (*n* = 166), log-Jacobian determinant maps (*n* = 165) and degree centrality (*n* = 129); please see Table S1. Exclusions for specific MRI analyses are depicted in Fig. S1.

Between-subgroup differences were investigated in the whole-brain at the voxel-level in terms of: log-Jacobian determinants, TBSS metrics (FA and MD) and degree centrality. FMRIB Software Library (FSL) [60] *randomise* function was used to implement non-parametric permutation methods for statistical inference. This method was used to model each contrast of interest for each voxel, i.e., a general linear model (GLM) correcting for PMA at scan and sex. rs-fMRI models also included motion estimates (standardized DVARS) as a covariate. Family Wise Error (FWE) rate with Threshold-Free Cluster Enhancement (TFCE) was applied to correct for multiple comparisons over the multiple voxels, while enhancing “cluster-like” structures of voxels without defining them as binary components [61]. Statistics were calculated using random permutation tests with 10000 permutations. Given the exploratory nature of our analysis, we did not correct for multiple contrasts tested (i.e., log-Jacobians, TBSS FA and MD, degree centrality). We show results significant at *p* < 0.05 FWE-corrected per contrast. Mean values from clusters of modality-specific voxels showing significant between-subgroup differences were extracted to calculate Cohen’s F effect sizes.

### Sensitivity analyses

There were 29 sets of children born from multiple pregnancy events in our sample. In order to account for multiple pregnancy confounding, we conducted additional sensitivity analyses including only one child from each set of multiple pregnancy siblings.

## Results

### Participant characteristics

Participants’ socio-demographic and clinical characteristics are shown in Table 1. Compared to participants who completed the follow-up assessment (*n* = 251; median GA = 29.24 weeks; median IMD at birth=19.48), individuals who were not assessed (*n* = 259; median GA = 29.27 weeks; median IMD at birth = 21.40) did not differ in GA (gr = 0.01; *p* = 0.807), but had greater neonatal socio-demographic deprivation (gr = 0.11; *p* = 0.028). Compared to the initial baseline cohort (n = 511; median GA = 30.00 weeks; median IMD at birth=18.19), participants who were studied here (*n* = 198) had slightly older GA (median GA = 30.14 weeks; gr = −0.13; *p* = 0.009) and relative socio-demographic advantage (median IMD score at birth=15.58, *gr* = 0.11; *p* = 0.027).

### Two-cluster solution subgroup profiles

When stratifying the sample into two clusters and comparing them in terms of in-model variables, subgroup 1 (termed here the ‘resilient’ subgroup) showed significantly better socio-communication (i.e., lower SRS-2 scores) and executive function abilities (i.e., lower BRIEF-P emotion control, inhibit, shift, working memory and plan/organize scores), lower emotion contagion (EmQue) scores, and higher prosocial actions scores (EmQue) during childhood, than subgroup 2 (termed here the ‘at-risk’ subgroup); all *p*s < 0.05, after FDR correction. The resilient subgroup had lower neonatal clinical risk compared to the at-risk subgroup, with a greater proportion of children receiving no neonatal mechanical ventilation and a smaller proportion of children receiving prolonged neonatal CPAP (both *ps* < 0.05, after FDR correction). Subgroups did not differ in terms of days on TPN in the neonatal period (*p* > 0.05).

**Fig. 2:**
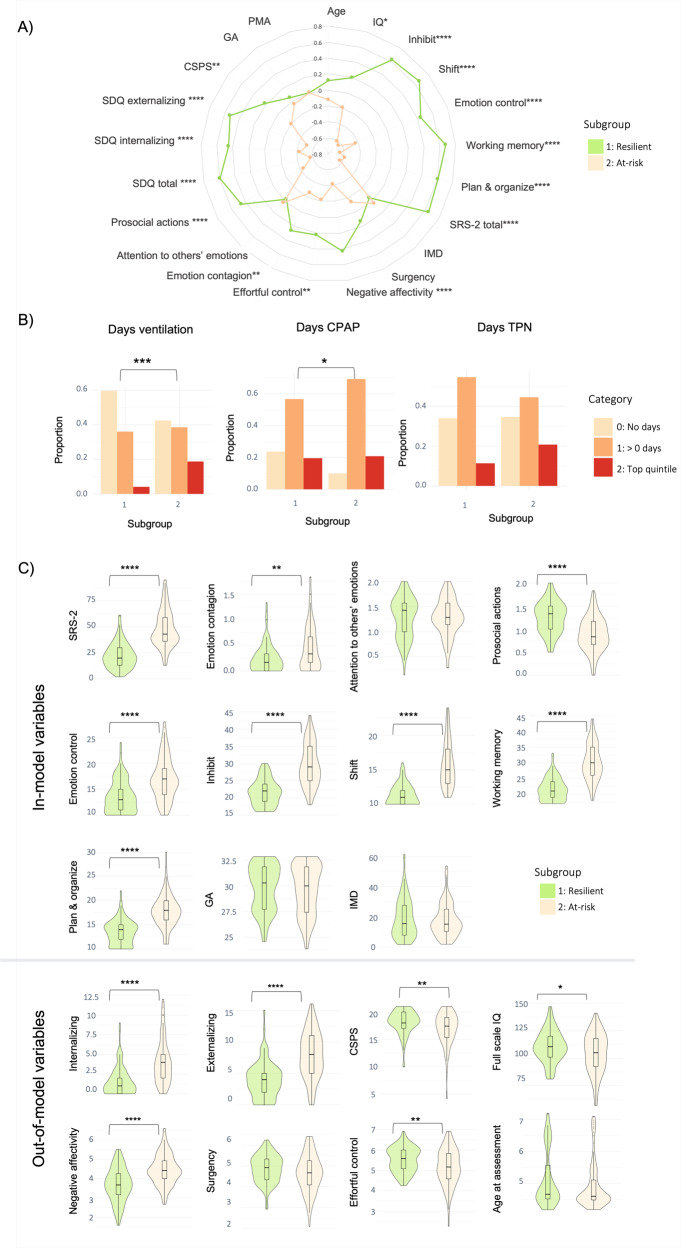
Two-cluster solution subgroup profiles. **A** Radar plot showing the two-cluster solution subgroup profiles using z-scores for subgroup 1 (i.e., resilient subgroup; green) and subgroup 2 (i.e., at-risk subgroup; beige). For visual illustrative purposes, scales which usually indicate poorer outcomes have been reversed so that larger z-scores on behavioral variables indicate better outcomes. **B** Bar plots for clinical risk variables (days on TPN, days on mechanical ventilation and days on CPAP, left to right, respectively) for each of the two subgroups. Plots represent the proportion of children belonging to each clinical risk category within a subgroup, where category 0 represents the lowest clinical risk (light beige; no days of clinical intervention), category 1 represents medium clinical risk (orange; more than 0 days of intervention but less than the top quintile), and category 2 represents the highest clinical risk (red; within the top quintile). **C** Violin plots showing differences between the subgroups in terms of in-model and out-of-model variables. Significant differences are marked with bars between the subgroups. *=*p* < 0.05; **=*p* < 0.01; ***=*p* < 0.001, ****=*p* < 0.0001.

Differences in out-of-model variables included lower psychopathology scores (SDQ internalizing and externalizing problems) and negative affectivity scores (CBQ) as well as higher effortful control (CBQ), IQ and cognitive stimulation at home (CSPS) during childhood in the resilient compared to the at-risk subgroup; all *p*s < 0.05, after FDR correction (Fig. 2; Table S2).

The two subgroups showed no significant differences in log-Jacobian determinant values, degree centrality or white matter microstructural characteristics (all *p*s > 0.05). Resultant subgroups also did not show differences in sex, age at assessment or PMA at scan (Fig. 2; Table S2).

### Three-cluster solution subgroup profiles

**Fig. 3:**
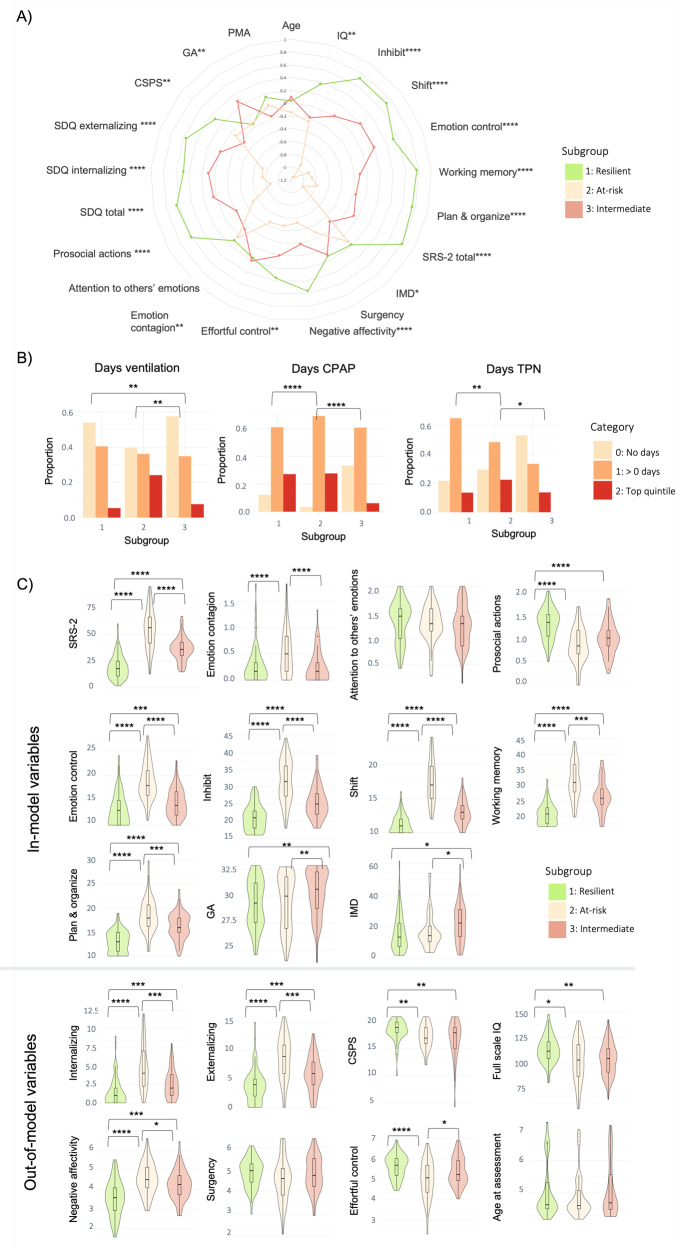
Three-cluster solution subgroup profiles. **A** Radar plot showing the three-cluster solution subgroup profiles using z-scores. For visual illustration purposes, scales which usually indicate poorer outcomes have been reversed, so that larger z-scores on behavioral variables indicate better outcomes. Subgroup 1 (resilient) is marked in green, subgroup 2 (at-risk) in beige and subgroup 3 (intermediate outcomes but lowest clinical risk) in pink. **B** Bar plots for clinical risk variables (days on TPN, days on mechanical ventilation and days on CPAP, left to right, respectively) for each of the three subgroups. Plots represent the proportion of children belonging to each clinical risk category within a subgroup, where category 0 represents the lowest clinical risk (light beige; no days of clinical intervention), category 1 represents medium clinical risk (orange; more than 0 days of intervention but less than the top quintile), and category 2 represents the highest clinical risk (red; within the top quintile). **C** Violin plots showing differences in in-model and out-of-model measures at the group-wise level. Significant differences are marked with bars between the subgroups. *=*p* < 0.05; **=*p* < 0.01; ***=*p* < 0.001, ****=*p* < 0.0001.

To increase subtyping resolution and explore latent heterogeneity not captured by a two-subgroup partitioning, the sample was further stratified into 3 subgroups. Two of the three resulting clusters largely reflected profiles similar to those from C = 2. The first was a ‘resilient’ subgroup (subgroup 1) with favorable childhood socio-communicative (SRS-2), empathy (EmQue) and executive function (BRIEF-P) outcomes in terms of in-model variables; low childhood psychopathology (SDQ internalizing and externalizing problems) and negative affectivity scores (CBQ) and high effortful control scores (CBQ), IQ and cognitive stimulation at home (CPSP) in terms of out-of-model variables. The second was an ‘at-risk’ subgroup (subgroup 2), with the poorest outcomes in terms of in-model variables (childhood socio-communication (SRS-2), empathy and executive function (BRIEF-P) scores), as well as out-of-model childhood psychopathology (SDQ), effortful control (CBQ) and negative affectivity measures (CBQ), combined with the highest neonatal clinical risk (Fig. 3; Table S3).

A third subgroup (labeled ‘intermediate’) emerged, which had poorer in-model and out-of-model childhood cognitive and behavioral scores when compared to the resilient subgroup, but better scores when compared to those of the at-risk subgroup. The intermediate subgroup also had the lowest neonatal clinical risk compared to both resilient and at-risk subgroups (Fig. 3; Table S3). The transition of subject classifications from the two- to the three-cluster solution is illustrated in an alluvial plot (Fig. S3).

In terms of environmental factors, the resilient subgroup had higher levels of childhood cognitive stimulation at home (CSPS) in comparison to both at-risk and intermediate subgroups, while the intermediate subgroup had higher neonatal socio-demographic risk (IMD) in comparison to both at-risk and resilient subgroups. All *p*s < 0.05 after FDR correction. The three subgroups did not differ in sex, age at assessment or PMA at scan.

**Fig. 4:**
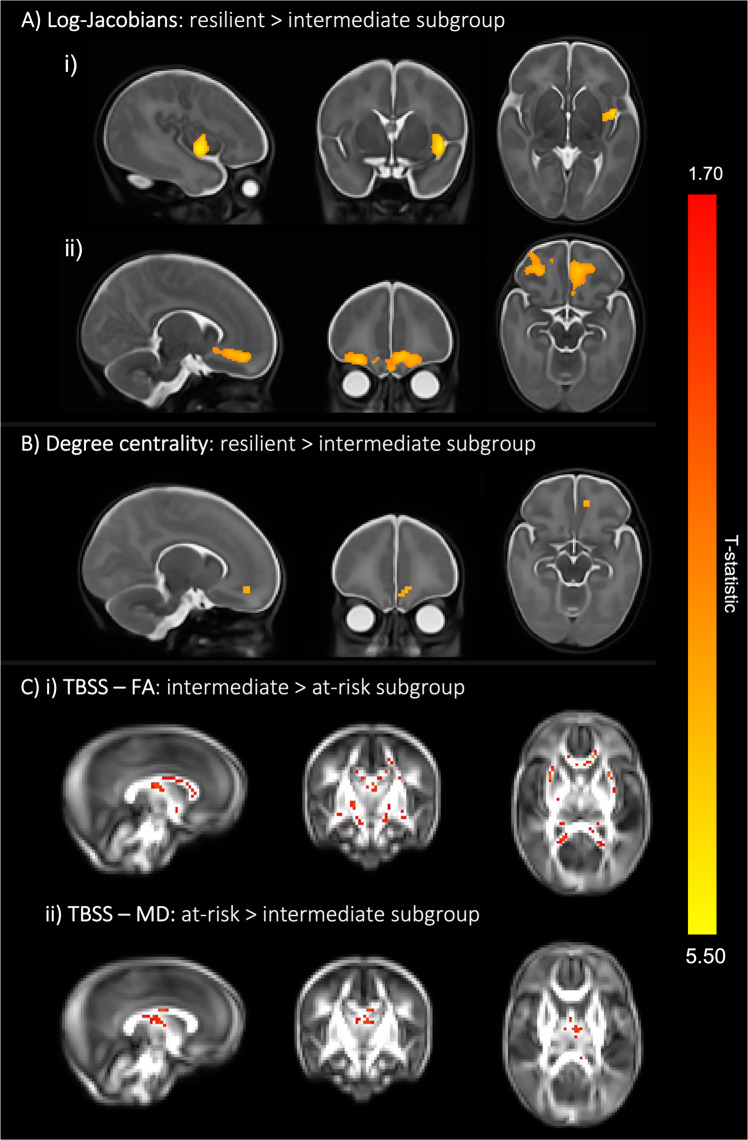
Three-cluster solution brain differences at term-equivalent age. **A** Colored voxels indicate regions with significantly larger log-Jacobian determinant values in the resilient subgroup (subgroup 1) compared to the intermediate subgroup (subgroup 3) in i) left insula and the ii) bilateral orbitofrontal cortices (*p* < 0.05). GLM included sex and PMA at scan as covariates and TFCE and FWE corrections were applied. **B** Voxels showing significantly larger degree centrality values in the resilient subgroup (subgroup) 1 compared to the intermediate subgroup (subgroup 3) are seen in an overlapping left orbitofrontal region at *p* < 0.05. GLM included sex, PMA at scan and motion (standardized DVARS) as covariates; TFCE and FWE were applied. **C** Colored voxels represent white matter regions showing i) significantly higher FA values in the intermediate subgroup compared to the at-risk subgroup and ii) significantly higher MD values in the at-risk subgroup compared to the intermediate subgroup (*p* < 0.05). T-statistic values are represented in the color bar, where red colored voxels indicate smaller T-statistic values and yellow voxels indicate higher T-statistic values ranging between 1.70 and 5.50.

In terms of brain imaging markers at term, the resilient subgroup displayed larger relative volumes (i.e., greater log-Jacobian determinant values) in the left insula and bilateral orbitofrontal cortices (Fig. 4A; Table S4) and higher degree centrality in an overlapping region in the left orbitofrontal cortex (Fig. 4B; Table S5) compared to the intermediate subgroup. The intermediate subgroup, compared to the at-risk subgroup, showed increased FA in several areas of the white matter skeleton, including the fornix, corpus callosum, corticospinal tract, inferior longitudinal, inferior fronto-occipital and uncinate fasciculi (Fig. 4Ci; Table S4), as well as lower MD in the fornix and body of the corpus callosum (Fig. 4Cii; Table S4). The resilient and at-risk subgroups did not differ in any brain measures (*p* > 0.05).

### Sensitivity analyses

Sensitivity analyses including only one sibling, selected at random from each multiple pregnancy set, revealed similar results (Table S5; Table S6; Fig. S6; Fig. S7), although the difference in neonatal functional connectivity between the resilient and intermediate groups was no longer significant (*p* = 0.08). In addition, the resilient subgroup displayed larger neonatal relative volume of the right insula compared to the intermediate subgroup. For more details, please refer to Supplemental Information.

## Discussion

Using an integrative clustering approach, we identified subgroups of VPT children with distinct neurodevelopmental profiles. We described a two-cluster solution, showing a resilient subgroup with comparably favorable childhood behavioral and cognitive outcomes and increased cognitive stimulation at home, and a second, at-risk subgroup, with poorer childhood behavioral and cognitive outcomes and high neonatal clinical risk. We also described a three-cluster solution, showing two subgroups largely characterized by the profiles observed in the two-cluster solution, as well as a newly emerging third intermediate subgroup, with a childhood behavioral and cognitive profile intermediate between the resilient and the at-risk subgroups. Nuanced differences in socio-demographic, neonatal clinical and early brain measures appeared upon comparing subgroups from the three-cluster solution. Notably, the resilient subgroup displayed larger fronto-limbic brain regions and increased functional connectivity at term compared to the intermediate subgroup. The at-risk subgroup showed widespread white matter microstructural alterations in fronto-temporo-limbic tracts compared to the intermediate subgroup. Furthermore, the resilient subgroup had a more cognitively stimulating childhood home environment compared to the at-risk and intermediate subgroups, while the intermediate subgroup had the lowest clinical risk. Together, these findings highlight the potential value of neonatal structural and functional brain measures as useful biomarkers of later childhood outcomes in distinct VPT subgroups, as well as the importance of a supportive home environment to foster child development.

In the at-risk subgroup from the two-cluster solution, poorer childhood socio-emotional, executive function, IQ, mental health and temperament outcomes may have been driven by a combination of both higher clinical risk at birth and a less stimulating childhood home environment, when compared to the resilient subgroup. Previous studies in VPT children have shown cognitively stimulating parenting to be positively associated with improved socio-emotional processing and cognitive outcomes at 2 years of age [62] and reduced psychopathology and executive function difficulties at 4–7 [54]. A cognitively stimulating home environment also differentiated between psychiatric profiles at 5 [8]. Moreover, increased neonatal clinical risk in the at-risk subgroup is consistent with previous findings, showing that perinatal medical complications following VPT birth may lead to increased behavioral and developmental problems [15, 16, 63]. The resilient and at-risk subgroups, however, did not differ in any of the neonatal brain measures investigated, suggesting that there may be additional non-measured variables underlying different childhood outcomes that need further investigation, such as alterations in pro-inflammatory immunomarkers [64, 65] and/or microbiome assembly [66, 67], which are reportedly associated with increased behavioral difficulties.

To further parse heterogeneity in VPT children, we also explored a three-cluster solution. These analyses showed that two subgroups mostly reflected the profiles seen in the two-cluster solution: 1) a resilient subgroup with high levels of childhood cognitive stimulation at home and 2) an at-risk subgroup with high levels of neonatal clinical risk. A third subgroup with intermediate childhood behavioral and cognitive profiles also emerged, in which childhood psychopathology, temperament and cognitive outcomes were poorer than those observed in the resilient subgroup, but more favorable than those observed in the at-risk subgroup. Intriguingly, the intermediate subgroup exhibited the lowest neonatal clinical risk compared to the other two subgroups, with a greater proportion of infants receiving no neonatal mechanical ventilation, CPAP or TPN and with higher median GA at birth. However, the intermediate subgroup also had higher environmental risk, namely reduced childhood cognitively stimulating home environment, compared to the resilient subgroup, and higher neonatal socio-demographic deprivation, compared to both the at-risk and resilient subgroups. These findings suggest that developmental outcomes may not be understood by exploring a single causal pathway and are best studied in a multidimensional space; for example, clinical risk, which has been linearly correlated with developmental outcomes in previous studies [16, 63], ought to be investigated together with other factors that may influence development, i.e., environmental risk.

The at-risk compared to the intermediate subgroup showed widespread alterations in white matter microstructure (lower FA and higher MD) in the fornix, corpus callosum, corticospinal tract, inferior longitudinal, inferior fronto-occipital and uncinate fasciculi. The at-risk subgroup had also the highest neonatal clinical risk, hence the observed white matter changes are likely to be associated with preterm-related neonatal complications [12, 68, 69]. White matter alterations in fronto-temporo-limbic tracts, including those observed here, have been previously associated with poorer cognitive outcomes [70–75]. They have also been implicated in emotion processing [76–78] and psychiatric disorders, including depression and schizophrenia [79–81]. The intermediate subgroup, conversely, had the lowest neonatal clinical risk, and higher FA/lower MD values in fronto-temporo-limbic tracts compared to the at-risk subgroup. These findings led us to speculate that having relative fewer neonatal clinical complications, and hence fewer preterm-related white matter alterations, may contribute to these children’s more favorable cognitive, socio-emotional and behavioral outcomes, compared to the at-risk subgroup.

Children in the resilient subgroup exhibited higher prosocial behavior and empathy, as well as fewer childhood externalizing and internalizing symptoms and executive function difficulties, compared to the intermediate and at-risk subgroups. They also showed lower childhood negative affectivity scores, referring to the expression of dysregulated negative emotions and increased sensitivity in response to surrounding stimuli [82, 83]. While the resilient group showed no significant brain differences compared to the at-risk subgroup, we speculate that the combination of two protective factors, an enriching home environment and lower neonatal clinical risk, may have contributed to attenuating the expression of the behavioral and cognitive difficulties associated with VPT birth. These findings also support the idea of multi-finality, whereby individuals with no overt brain differences at term may display distinct behavioral outcomes later in childhood.

Compared to the intermediate subgroup, however, the resilient subgroup displayed larger relative volumes in the left insular and bilateral orbitofrontal cortices and increased functional connectivity in an overlapping left orbitofrontal region at term, years before the behavioral and cognitive childhood outcomes were assessed. These findings could be interpreted in terms of a more advanced maturation of the fronto-limbic network in the resilient subgroup, as orbitofrontal functional connectivity and insular cortical microstructure and morphology have been positively associated with GA at birth and PMA at scan [84–86]. However, as several other brain areas are undergoing rapid neurodevelopmental changes at the time our participants underwent MRI, including somatosensory, occipital, temporal, parietal and other areas of frontal cortex [86], we speculate the orbitofrontal cortex and the insula may be preferentially discriminating between the intermediate and the resilient subgroup, in the context of the brain-wide analysis approaches employed here, because they play critical functional roles in the cognitive and behavioral outcomes we studied. The orbitofrontal cortex is involved in the top-down regulation of goal-oriented executive functions and socio-emotional processing, reward-guided learning and decision making [87–89]; the insula is important for regulating internal processes, including emotional responses to external stimuli [90]. Structural alterations in the orbitofrontal cortex and insula, which are structurally connected [91], have been associated with emotion dysregulation [92] and with higher externalizing behaviors [93].

The orbitofrontal cortex is sensitive to environmental stimuli, such as early life stress [94, 95]. Individuals with a history of physical abuse [96] and VPT infants exposed to painful procedures [97] both show reduced orbitofrontal volumes in childhood. Furthermore, alterations in orbitofrontal connectivity and gyrification have been associated with social processing impairments in VPT children [98] and with executive function difficulties in extremely preterm (EPT; < 28 weeks’ gestation) adolescents [99], respectively. Smaller insular volumes have been associated with worse emotion regulation skills [100] and weaker insular functional connectivity with decreased empathic responses [101]. In the late preterm period, the insula becomes a key hub region [102] and a major source of transient bursting events that support brain maturation [103]. A more mature fronto-limbic network may have therefore supported a favorable development of emotion regulation capacity, cognition, and behavior [104, 105], resulting in the resilient subgroup exhibiting lower externalizing and internalizing symptoms, increased empathy, emotion regulation abilities and executive function skills in childhood.

This study demonstrates that it is possible to parse heterogeneity in VPT children in a meaningful way. We show that protective brain maturational patterns in the neonatal period may contribute to a more resilient behavioral profile in childhood. This is encouraging, as the preterm brain is susceptible to neuroplastic changes in response to behavioral and environmental interventions, both early in life and later in childhood [106]. For example, neuroplastic changes have been observed following ‘supportive-touch’ (i.e., skin-to-skin contact or breastfeeding’) [107], maternal sensitivity training [108], visual stimulus cues of the mother’s face [109], parental praise [110] or music interventions in the neonatal intensive care unit [111]. Such methods could, therefore, be used in the future to strengthen fronto-limbic circuitry to boost children’s resilience. Furthermore, our findings suggest that enriching environments may promote resilience towards more favorable behavioral outcomes. This could be done by increasing parental awareness about the importance of cognitive stimulation at home. Our findings also show that the subgroup of children with the highest neonatal clinical risk exhibit the poorest outcomes, highlighting the need to develop and implement targeted interventions for the most clinically vulnerable VPT children.

It is worth noting that the median outcome scores (IQ, BRIEF-P, SRS-2 and SDQ) for our three subgroups were within normative ranges and below clinical thresholds, even for the at-risk subgroup. Subthreshold psychiatric symptoms have been reported in other at-risk subgroups of VPT children [9, 8], and have also been associated with an increased risk of developing psychiatric disorders later in life [112]. In this context, subthreshold psychiatric symptoms may represent transdiagnostic traits that would remain undetected, and therefore untreated, if considered in a purely clinically diagnostic context, highlighting the importance of addressing psychopathology dimensionally [113, 114].

Strengths of this study include a fairly large sample size and a rich longitudinal dataset with clinical data from birth, neonatal multi-modal MRI at term and behavioral follow-up in early childhood. However, a limitation of this study is that the VPT participants included in our analyses (*n* = 198) had a relative socio-demographic advantage and older gestational age at birth than the initial baseline cohort (*n* = 511), which may limit the generalizability of our findings to a portion of the socio-demographic and gestational age spectrum. In addition, the lack of a full-term group and the exclusion of children with major brain lesions in the integrative-clustering analyses may have also limited the variability in our data, and in turn contributed to the failure to identify a more impaired subgroup here. Future studies must take extra caution when interpreting such results and make increased efforts to recruit more diverse participant samples.

Additional limitations to consider include the use of parental reports for most child behavioral measures, except IQ, which could lead to common method variance bias [115] and result in underreporting of psychopathology [116]. The lack of information on familial cognitive outcomes and psychiatric history, which are heritable traits [117], prevents us from estimating trait heritability. Moreover, the small to moderate effect sizes reported for neonatal brain differences between subgroups may limit their immediate clinical meaningfulness or translatability into clinical practice. However, the fact that these brain differences only emerged after subdividing the sample into more refined and homogenous phenotypic subgroups (C = 3 vs C = 2), highlights the benefit of using advanced clustering approaches such as SNF. We speculate that these effects may be diluted in the two-cluster solution due to the presence of individuals within both (at-risk and resilient) subgroups having profiles that are more similar to an intermediate subgroup profile (please see Fig. S3).

Sensitivity analyses including one sibling only from each twin/triplet set mostly replicated the main findings, showing similar early brain patterns as well as cognitive, neonatal clinical, social, and childhood behavioral profiles for both two- and three-cluster solutions, suggesting that the effects seen here are not biased by the presence of multiple pregnancy siblings in the main analyses. While the functional connectivity results were no longer significant in the sensitivity analyses, we speculate this may be due to a loss in power associated with the reduced sample size.

In summary, using an integrative clustering approach, we were able to stratify VPT children into distinct multidimensional subgroups. A subgroup of VPT children at risk of experiencing behavioral and cognitive difficulties was characterized by high neonatal clinical complications and white matter microstructural alterations at term, whereas a resilient subgroup, with comparably favorable childhood behavioral outcomes, was characterized by increased childhood cognitive stimulation at home and larger and functionally more connected fronto-limbic brain regions at term. These results highlight a potential application of precision psychiatry, to enable meaningful inferences to be made at the individual level. Patterns of fronto-limbic brain maturation may be used as image-based biomarkers of outcomes in VPT children, while promoting enriching environments may foster more optimal behavioral outcomes. Risk stratification following VPT birth could, therefore, guide personalized behavioral interventions aimed at supporting healthy development in vulnerable children.

## Supplementary information

Supplemental Information document

## Data Availability

Access to the dataset supporting this article can be made available upon request from the corresponding author. Code used to generate the results central to this paper can be accessed here: https://github.com/lailahadaya/preterm-ExecuteSNF.CC.
